# Effects of high moisture ear corn on production performance, milk fatty acid composition, serum antioxidant status, and immunity in primiparous dairy cows

**DOI:** 10.5713/ab.23.0318

**Published:** 2024-04-01

**Authors:** Songlin Shang, Zheng Li, Jiajun Li, Xi Zhao, Wenjing Zhang, Xinrui Zhang, Jinni Bai, Zhiye Yang, Kaijun Guo

**Affiliations:** 1 College of Animal Science and Technology, Beijing University of Agriculture, Beijing 102206, China; 2 Beijing Institute of Feed Control, Beijing 100107, China

**Keywords:** Antioxidant Capacity, Dairy Cows, High Moisture Ear Corn, Immunity, Production Performance

## Abstract

**Objective:**

This study evaluated the effects of high moisture ear corn (HMEC) on production performance, milk fatty acid composition, serum antioxidant status, and immunity in primiparous dairy cows.

**Methods:**

A total of 45 healthy primiparous Holstein cows (36.50±4.30 kg of milk/d, 201±9.00 lactating days in milk) were sorted into 3 groups: control group (CG, n = 15); 50% HMEC (replacing 50% steam-flaked corn with HMEC, n = 15); and 100% HMEC (replacing steam-flaked corn with HMEC, n = 15) on an equal dry matter (DM) basis. The study consisted of adaptation period of 14 days, followed by a formal period of 60 days. Feed intake and milk yield were recorded daily. Milk and blood samples were collected on 1, 30, and 60 d of the experimental period.

**Results:**

The 50% HMEC group and 100% HMEC group significantly increased (p<0.05) milk yield and DM intake in dairy cows compared to the control group (CG). The 100% HMEC group showed an increase (p<0.05) in 4% fat-corrected milk (4% FCM). Both the 50% HMEC group and 100% HMEC group exhibited significant decreases (p<0.05) in the content of C10:0, C12:0, and C14:0 fatty acids, along with a significant increase (p<0.05) in cis-9C18:1 content. The saturated fatty acid content was significantly lower (p<0.05) in the 50% HMEC and 100% HMEC groups than that of CG. Conversely, the monounsaturated fatty acid content was higher (p<0.05) in the 50% HMEC and 100% HMEC groups than that in CG. Notably, the 100% HMEC group significantly increased (p<0.05) the serum superoxide dismutase and glutathione peroxidase content, while also decreasing the serum malondialdehyde content (p<0.05). Moreover, the 100% HMEC group significantly increased (p<0.05) the content of immunoglobulin G (IgG) and IgM.

**Conclusion:**

High moisture ear corn could improve production performance and milk fatty acid levels and enhance immunity and antioxidant capacity in dairy cows. These results lay the foundation for the wider application of HMEC in ruminant animal diets.

## INTRODUCTION

Corn, as the primary source of energy feed, is commonly processed and stored in two ways: ground and steam flaked. Moisture presents a challenging factor during corn storage, as improper storage can lead to a reduction in corn’s nutritional composition and the accumulation of aflatoxin. High moisture corn (HMC) effectively avoids this problem by fermentation under anaerobic condition, mitigating the risk of mold development, and thereby maintaining the nutritional integrity of the corn while preventing aflatoxin accumulation. On the other hand, HMC undergoes specific operations such as grinding, compaction, and sealing within a wet storage silo, which results in a decreased oxygen density. This environment promotes the dominance and substantial proliferation of lactic acid bacteria, utilizing sugars and water-soluble carbohydrates in the raw material to produce lactic acid, effectively lowering the pH to a range of 3.7 to 4.1. Consequently, yeast, mold, aerobic bacteria, and other harmful microorganisms are inhibited, providing mold inhibition and preservation effects [1]. HMC is rich in lactic acid and acetic acid after milling and fermentation, and it also has a great protective effect on β-carotene content [2]. Due to its high starch and fat content and small starch granules after fermentation, HMC is a high starch energy feed [3]. Rojas-Garduño et al [4] demonstrated that feeding HMC (3.5 kg DM/cow/d) significantly increased milk production and enhanced the concentration of unsaturated fatty acids in milk. Furthermore, it was discovered that feeding HMC can lead to increased ruminal starch digestion rate, feed efficiency, and microbial protein production [5,6].

The economic significance of primiparous cows in livestock farming cannot be ignored. Miglior et al [7] emphasized the positive correlation between the genetic quality of primiparous cows and the overall profitability of dairy operations. Their potential to contribute to a more efficient and productive herd directly impacts the bottom line. HMC has gained recognition for its nutritional richness, capacity to boost milk production, and ability to reduce feed costs [8]. High moisture ear corn (HMEC) is a specialized fermentation of corn cobs and kernels. This approach augments its nutritional profile, resulting in a notably elevated content of neutral detergent fiber in comparison to conventional HMC and can also increase biological yield. This enhancement bears potential advantages in averting ruminal acidosis in dairy cows. Nonetheless, there is little research dedicated to the investigation of HMEC. This study aims to explore the impacts of varying levels of HMEC as a substitute for steam-flaked corn on milk production performance, milk fatty acid composition, serum antioxidant status, and immunity in primiparous dairy cows This would provide a scientific theoretical basis for applying HMEC in dairy cow production.

## MATERIALS AND METHODS

### High moisture ear corn

The HMEC utilized in this study, manufactured by Qiushi Grain Trade Company (Xingtai, Hebei Province, China), consisted of corn Weko 702, encompassing both corn kernels and corn cobs, harvested at the fully ripened stage with an initial moisture content ranging from 28% to 32%. Following the crushing of HMEC using a grinder, it was thoroughly mixed with additives and subjected to 60 days wrapping fermentation process. The main ingredients and contents on a dry matter (DM) basis were as follows: DM 63.48%, crude protein 6.53%, crude fat 3.13%, ash 1.35%, neutral detergent fiber 13.17%, acid detergent fiber 2.97%, and starch 59.57%. The fermentation characteristics of HMEC are delineated as follows: pH 3.87, lactic acid (% DM) 2.76, acetic acid (% DM) 0.58, and NH3-N (% TN) 14.87.

### Animals

The Ethics Committee on Animal Use at Beijing University of Agriculture approved the procedures for animal care and handling necessary for this experiment (Beijing, China) (approval number: BUA2023072). A total of 45 healthy primiparous dairy cows (36.50±4.30 kg of milk/d, 201±9.00 lactating days in milk) were sorted into 3 groups: control group (CG, n = 15), 50% HMEC (replacing 50% steam-flaked corn with HMEC, n = 15), and 100% HMEC (replacing steam-flaked corn with HMEC, n = 15) on an equal DM basis. The diets were formulated according to the standards provided by the National Academies of Sciences, Engineering and Medicine (NASEM, 2021) [9]. The composition and nutrient content of the diets can be found in Table 1. The study consisted of an adaptation period of 14 days, followed by a formal period of 60 days. Throughout the experimental period, all animals were provided ad libitum with feed and fresh water. The dairy cows were milked triple per day at 02:00, 11:00, and 20:00, respectively.

### Feed intake sampling, milk sampling and analysis

Feed intake and milk yield were documented daily. To guarantee complete intake, the dairy cows were provided with unrestricted access to the diet. To calculate dry matter intake (DMI), feed quantities for stall barn cows were measured, alongside the remaining feed, three times a week and finally averaged.

Raw milk samples, approximately 50 mL in volume, were collected from each dairy cow three times a day, following morning, afternoon, and evening milking, on days 1, 30, and 60, respectively. The first 20 mL of collected milk at each time was discarded, and the milk samples were mixed according to the ratio of 4:3:3 (vol/vol, morning: afternoon: evening) daily. Thoroughly mixed milk samples were subsampled daily and mixed by cows after total collection. Mixed milk was preserved with saturated potassium dichromate solution at 4°C for milk fat, protein, lactose, total solids, and urea determination by an infrared milk analyzer (Delta FT-ADelta FT-A, PerkinElmer, Nederland) within 2 to 4 d after sampling. The 4% fat-corrected milk (FCM) and feed efficiency was calculated using the following equation: 4% FCM (kg/d) = (0.4+ [0.15×milk fat (%)])×milk yield (kg/d). Feed efficiency = 4% FCM/DMI.

### Milk fatty acid analysis

The method of Bradford et al [10] was used for the determination of fatty acids in milk, by a gas chromatography-mass spectrometry coupled instrument (Agilent Technologies Ltd., Santa Clara, CA, USA), chromatographic conditions: HP-5 flexible quartz capillary column (300 m×25 mm×0.25 μm), inlet temperature of 280°C, injection volume of 0.2 μL, shunt ratio. The sample volume was 0.2 μL, and the split ratio was 20:1. The mass spectrometry (MS) conditions were as follows: the ion source was an electronic ionization source, the ionization voltage was 70 eV, the temperature of the ion source was 280°C, the emission current was 34.6 mA, and the scanning range was 30 to 500 amp.

### Blood collection and sampling analysis

During the experimental period, blood samples were collected from the jugular vein of all dairy cows prior to their morning feeding on days 1, 30, and 60. For blood collection, 10 mL Vacutainer tubes without any additives (Shandong aosaite Medical Instrument Co., Ltd., Shandong, China) were utilized, resulting in the extraction of approximately 8 mL of blood from each cow. Subsequently, the samples were centrifuged at 3,000×g for 15 minutes at 4°C, and the resulting serum was carefully transferred into 2 mL tubes. The serum samples were then stored at −20°C until they were ready for analysis.

The levels of glucose (GLU), cholesterol (CHOL), albumin (ALB), alanine aminotransferase (ALT), aspartate aminotransferase (AST), urea (UREA), creatinine (CREA), and triglyceride (TG) in the serum were assessed using an automated biochemical analyzer (TBA-120FR; TOSHIBA Ltd., Tokyo, Japan). The levels of total antioxidant capacity (T-AOC, A015-2), superoxide dismutase (SOD, A001-3-2), glutathione peroxidase (GSH-Px, A005-1-2), catalase (CAT, A007-1-1), malondialdehyde (MDA, A003-1-2), immunoglobulin M (IgM, E025-1-1), immunoglobulin G (IgG, E026-1-1), immunoglobulin A (IgA, E027-1-1), and tumor necrosis factor-α (TNF-α, H052-1-2) in the serum were assessed using commercial ELISA kits (Nanjing Jiancheng Bioengineering Institute, Nanjing, China) according to the manufacturer’s instructions, respectively.

### Data analysis

Data on milk yield and composition, DMI, milk fatty acid and serum indices were analyzed using the MIXED procedure in SAS (ver. 9.4, SAS Institute Inc., Cary, NC, USA). The statistical model included treatment and sampling date as the fixed effects, sampling date as the repeated measurement, and dairy cows as the random effect. Contrasts were constructed to examine treatment effects and replaces HMEC levels, with orthogonal polynomials accounting for the unequal spacing of HMEC levels. The differences between the groups were evaluated by Tukey’s multiple range test, and the data were presented as least squares means and standard errors of mean. All analyses were considered significant at p<0.05, and trends at 0.05≤p<0.10 were discussed.

## RESULTS

### Feed intake, milk yield and composition

A linear increase in milk yield (p<0.02) and DMI (p<0.01) was observed, with the highest level recorded in the group fed on 100% HMEC (Table 2). In addition, the 4% FCM (p< 0.02) were higher in cows fed on 100% HMEC relative to those in the CG and 50% HMEC group. However, there were no significant differences for milk composition among groups (p>0.05).

### Milk fatty acid

Table 3 presents the effects of feeding HMEC on milk fatty acids in dairy cows. Both 50% HMEC and 100% HMEC groups, significantly decreased (p<0.05) the content of C10:0, C12:0, and C14:0, and significantly increased (p<0.05) cis-9C18:1 content. The saturated fatty acid (SFA) content was significantly lower (p<0.05) in 50% HMEC and 100% HMEC group compared to those in CG. Conversely, the monounsaturated fatty acid (MUFA) content was significantly higher (p<0.05) in the 50% HMEC and 100% HMEC group than CG. However, there was no significant difference in the content of other fatty acids among the groups (p>0.05).

### Biochemical indices in serum

The effect of feeding HMEC on the biochemical indices of dairy cows is shown in Table 4. The results indicated that there were no significant differences on ALT, AST, ALB, GLU, UREA, CREA, CHOL, TG, NEFA, T3, and T4 among the groups (p>0.05).

### Antioxidant status in serum

As presented in Table 5, when compared to those in CG, the 50% HMEC group had no significant differences in their antioxidant status (p>0.05). However, 100% HMEC group had increased (p<0.05) serum SOD and GSH-Px content, while also significantly lower (p<0.05) serum MDA content. There were no significant differences on T-AOC and CAT in 100% HMEC group (p>0.05).

### Immune responses in serum

As observed in Table 6, in comparison to those in CG, 50% HMEC had no significant differences in their immune responses of the serum (p>0.05). Remarkably, 100% HMEC group significantly increased (p<0.05) the content of IgG and IgM in the serum. However, IgA and TNF-α did not differ in 100% HMEC group (p>0.05).

## DISCUSSION

DMI is a crucial factor influencing the performance and health status of lactating dairy cows and can impact milk production [11]. In this study, both 50% HMEC group and 100% HMEC group had significantly increased DMI, which is similar to the findings of a previous study [12]. This might be attributed to the fact that the HMEC fed to the cows had a fermented aromatic odor, soft texture, and was easily digestible, which enhanced its palatability. During the fermentation of HMEC, a substantial quantity of lactic acid is generated, which is further fermented to produce ethanol. Moreover, in our subsequent studies, our team observed that *L. citreum* is the predominant genus in HMEC, which has the capability of breaking down 2,3-butanedione, acetic acid, and ethanol, all of which play a role in shaping the product’s flavor profile. Milk production is a paramount indicator used to evaluate the performance of dairy cows, and it is influenced by various factors, with ration nutrition being one of the most significant [13]. Rojas-Garduño et al [4] found that feeding HMC (3.5 kg DM/cow/d) to dairy cows led to a significantly increased milk production of 2.3 kg/d. In the present study, the milk yield significantly increased in the 50% HMEC group and the 100% HMEC group by 0.81 kg and 1.18 kg, respectively, which similar with previous research findings. This might be attributed to the breakdown of the hydrophobic starch-protein matrix surrounding the starch granules after HMEC entered the dairy cow’s rumen, leading to more microbial fermentation of starch. This, in turn, increased the starch digestibility of HMEC in the rumen and intestinal tract of the cows, improving the energy supply for milk production and resulting in a significant milk yield increase.

Milk composition is a vital indicator of dairy cow performance, with milk fat, milk protein, and lactose being commonly used to assess the raw milk’s quality [14]. Bradford et al [10] reported feeding HMC to dairy cows (32.1% DM) showed no significant impact on milk fat, protein, and lactose in milk. These findings are similar with the results of the present study. Milk urea nitrogen content reflects the protein level in the ration. High urea concentration in milk indicates high rumen ammonia concentration, and excessive ammonia passes from the rumen into the bloodstream, being converted to urea in the liver [15]. However, the milk urea nitrogen content of all groups in this study was not significantly different. These results suggest that replacing steam-flaked corn with HMEC had no adverse effect on the health status of dairy cows under the study conditions. Moreover, HMEC is widely used all over the world and has a very good performance unless the HMEC is of poor quality or has rotted during fermentation.

In general, there are two main sources of fatty acids in milk. Firstly, they come from short and medium-chain fatty acids, which are derived from de novo synthesis in the mammary gland from rumen fermentation products like acetic acid and β-hydroxybutyric acid (BHBA). Secondly, fatty acids come from long-chain fatty acids primarily taken up by the mammary gland from the bloodstream [16]. In the present study, both 50% HMEC group and 100% HMEC group showed a significant reduction in the content of C10:0, C12:0, and C14:0 fatty acids, which was similar to the findings of Rojas-Garduño et al [4]. The reason for this similarity could be attributed to the inhibition of de novo synthesis in the mammary gland, as the increased presence of long-chain fatty acids in HMEC might have inhibited acetyl coenzyme A activity. Additionally, the lower fatty acid concentrations may be due to the utilization of acetic acid and BHBA from rumen-fermented carbohydrates in the mammary gland to synthesize C4:0-C14:0 fatty acids [17]. Moreover, there was a significant increase in cis-9C18:1 content in the 50% HMEC and 100% HMEC groups, which could be attributed to HMEC being a supplement with higher concentrations of cis-9C18:1 and C18:2n-6 long-chain fatty acids. These long-chain fatty acids can increase the flow of C18 fatty acids into the duodenum, thus enhancing the synthesis of long-chain fatty acids in milk [10]. In this study, feeding HMEC resulted in lower concentrations of SFA and higher concentrations of MUFA. Previous literature has shown that feeding HMEC helps cows consume polyunsaturated fatty acid (PUFA) precursors, leading to an increase in the content of PUFA in the milk [4], which similar with the present findings. This difference in fatty acid content between steam-flaked corn and HMEC, along with the combined effect of the rumen environment and different rations, could have influenced rumen biohydrogenation and, subsequently, the fatty acid content of the milk.

Serum biochemical indexes play a crucial role in reflecting the nutritional level, metabolic status, growth, development, and production performance of dairy cows [18]. ALT and AST are relatively important transaminases in ruminants, and not only are they related to protein metabolism, but their activity levels can also reflect liver-related functions. ALT and AST are important indicators reflecting the degree of liver damage and liver cell damage and can reflect the permeability of the hepatocyte membrane [19]. ALB helps maintain blood osmotic pressure and transports nutrients and proteins in the body. GLU regulates the animal’s vital activities through oxidative energy release or conversion [20]. Aleixo et al [21] fed cows with HMC and sugar beet silage separately and found no significant difference in ALB and GLU levels in the serum of cows in the HMC feeding group, indicating that HMC did not have any adverse effects on the cows. In the present study, feeding HMEC had no significant effect on serum levels of AST, ALT, ALB, and GLU in dairy cows, which is similar to the results of the previous study. This suggests that HMEC does not have any adverse effects on the nutritional and metabolic levels of dairy cows.

Under normal physiological conditions, the free radical content in the animal’s body is in a state of dynamic equilibrium. When this equilibrium is disrupted, a large number of free radicals will be generated, leading to oxidative stress, which can adversely affect the production performance of dairy cows. The animal’s antioxidant defense system includes enzymes like SOD, CAT, GSH-Px, etc [22]. SOD acts as the first line of defense against oxidative stress by catalyzing the conversion of superoxide radicals into hydrogen peroxide and can further catalyze reactive oxygen species (ROS) to generate H_2_O_2_ and O_2_ [23]. GSH-Px then decomposes H_2_O_2_ to generate harmless products like H_2_O and O_2_, thereby protecting cells from oxidative stress damage [24]. T-AOC is a measure of the total capacity of the organism to scavenge reactive oxygen/nitric oxide species (ROS/NOS), which can indicate the degree of oxidative stress in the organism [25]. MDA is a product formed after the peroxidation reaction of free radicals attacking biological membranes, and its content indicates the degree of lipid peroxidation [26]. Albornoz et al [27] found no significant difference in any of the antioxidant indices of cows fed HMC with different starch contents, indicating no effect on the antioxidant capacity of the lactating cow. However, in the present study, 100% HMEC group showed an increase in serum levels of SOD and GSH-Px and a decreased serum level of MDA, suggesting stress relief and enhanced antioxidant capacity. This difference in results could be related to the fact that HMEC produces abundant amino acids, and lactic acid bacteria during wet storage [28]. Lactic acid bacteria have been shown to display antioxidant capacity by secreting their own hydrophobic alcohols like glutathione and antioxidant enzymes like SOD to resist oxidative stress [29]. Thus, under the conditions of the present study, replacing steam-flaked corn with 100% HMEC may improve the antioxidant capacity of dairy cows.

TNF-α, mainly synthesized and secreted by activated monocyte macrophages, is one of the earliest and most dominant inflammatory factors in the body’s anti-infective response [30]. It plays a potent pro-inflammatory cytokine role in animals and has immunosuppressive effects in autoimmune diseases [31]. Albornoz et al [27] found that the serum TNF-α levels between cows fed HMC and dry maize in their ration were not significantly different, indicating that feeding HMC did not induce an inflammatory response in cows. Similarly, in the present study, the serum TNF-α levels in the groups showed no significant differences, suggesting that replacing pressed maize with HMEC does not cause an inflammatory response in dairy cows. IgM, IgG, and IgA play crucial roles in specific immunity, and an increase in their levels enhances the immune function [32]. IgM is the first immunoglobulin that generates an immune response in humoral immunity when the body is stimulated by antigens, playing a vital role in the early immune defense. IgG constitutes about 75% of the total immunoglobulin in the serum, and it is the most important and abundant antibody in the body, possessing antiviral, antimicrobial, and immunomodulatory functions. IgA acts as the first line of defense against pathogens. Wang et al [33] administrated HMC to weaned piglets, and observed that HMC supplementation led to a notable reduction in coliforms within the intestinal tract, consequently enhancing the immune response of the piglets. In the present study, 100% HMEC group had increased levels of IgG and IgM in the serum, similar with previous research findings. This could be attributed to several factors. Firstly, the production of organic acids like lactic acid and acetic acid after wet storage of HMEC can lower the gastrointestinal tract’s pH, inhibiting harmful bacteria activities, promoting beneficial bacteria growth, and increasing the secretion of antibodies in mesenteric lymph nodes, thereby enhancing cows’ immune abilities. Lactic acid bacteria can increase immunoglobulin concentration by boosting killer cells’ and macrophages’ activity, thus enhancing body immunity. Secondly, HMEC preserves β-carotene in maize, a precursor substance of vitamin A, which significantly increases antioxidant power and immunity in ruminants [34]. These factors collectively suggest that HMEC can improve the immunity of dairy cows.

## CONCLUSION

Replacing steam-flaked corn in the diet with equal DM basis HMEC resulted in notable improvements in the daily DMI, milk yield, 4% FCM and milk fatty acid levels of primiparous dairy cows, while leaving milk composition unaffected. Furthermore, at 100% substitution, elevated IgM, and IgG levels, along with increased SOD and GSH-Px levels, while reduced MDA levels in the serum of primiparous dairy cows. These findings indicated that incorporating HMEC into the diet could enhance the immunity and antioxidant capacity of primiparous dairy cows. These findings offered a valuable theoretical foundation for the application of HMEC in the ruminant production.

## Figures and Tables

**Table 1:** Composition and nutrient composition of the experimental diets (% of DM basis)

Items	Treatments^1)^		

	CG	50% HMEC	100% HMEC
Corn silage	15.77	15.77	15.77
Wheat silage	6.55	6.55	6.55
Alfalfa	12.01	12.01	12.01
Oats	1.72	1.72	1.72
Whole cotton seed	4.49	4.49	4.49
Beet granules	0.11	0.11	0.11
Brewers grain	10.94	10.94	10.94
High moisture ear corn	-	3.52	7.07
Steam flake corn	7.07	3.55	-
Corn grain	19.16	19.16	19.16
Soy bean meal	10.83	10.83	10.83
Expanded soybean	0.74	0.74	0.74
Canola meal	3.24	3.24	3.24
DDGS	3.83	3.83	3.83
Premix^2)^	1.27	1.27	1.27
Bypass fat	2.27	2.27	2.27
Nutritional levels			
NE_L_ (MJ/kg)^3)^	6.71	6.71	6.72
DM	49.50	49.50	49.50
CP	16.68	17.05	17.11
EE	5.21	5.90	5.89
ADF	15.12	15.13	15.35
NDF	25.33	27.71	28.11
Ash	7.95	7.80	7.85
Starch	25.31	25.11	25.05
Ca	0.69	0.69	0.69
P	0.38	0.39	0.38

HMEC, high moisture ear corn; DDGS, Distillers’ dried grains with solubles; NE_L_, net energy for lactation; DM, dry matter; CP, crude protein; EE, ether extract; ADF, acid detergent fiber; NDF, neutral detergent fiber.

1) CG, control group (n = 15); 50% HMEC, replacing 50% steam-flaked corn HMEC (n = 15); 100% HMEC, replacing steam-flaked corn with HMEC (n = 15).

2) The premix provided the following per kg of the diet: vitamin A 200,000 IU, vitamin D 40,000 IU, vitamin E 2,000 IU, Cu 180 mg, Zn 1,000 mg, Mn 800 mg, I 15 mg, Se 10 mg.

3) NE_L_ was a calculated value according to National Academies of Sciences, Engineering and Medicine (NASEM, 2021), while the others were measured values.

**Table 2:** Effect of HMEC on production performance in primiparous dairy cows

Items	Treatment^1)^			SEM	p-value^2)^		
	
	CG	50% HMEC	100% HMEC		T	L	Q
Milk yield (kg/d)	36.15^c^	36.96^b^	37.33^a^	0.23	0.01	0.02	0.48
DMI (kg/d)	22.23^c^	22.86^b^	23.83^a^	0.19	0.02	0.01	0.26
4% FCM (kg/d)	34.85^b^	36.29^ab^	36.77^a^	1.33	0.02	0.64	0.31
Feed efficiency	1.57	1.59	1.54	0.06	0.33	0.54	0.42
Fat (%)	3.76	3.88	3.90	0.34	0.21	0.31	0.25
Protein (%)	3.22	3.32	3.33	0.20	0.83	0.50	0.34
Lactose (%)	5.14	5.35	5.37	0.22	0.54	0.12	0.66
Total solid (%)	13.35	12.96	12.92	0.74	0.81	0.34	0.54
Milk urea nitrogen (mg/dL)	13.35	13.43	13.53	1.17	0.99	0.41	0.41

HMEC, high moisture ear corn; SEM, standard error of the mean; DMI, dry matter intake; FCM, fat-corrected milk.

1) CG, control group; 50% HMEC, replacing 50% steam-flaked corn with high moisture ear corn; 100% HMEC, replacing 100% steam-flaked corn with high moisture ear corn.

2) T, treatment; L, linear; Q, quadratic.

a–c Different lowercase superscripts within the same row indicate significant differences (p<0.05).

**Table 3:** Effect of HMEC on milk fatty acid in primiparous dairy cows (g/100 g FA)

Items	Treatment^1)^			SEM	p-value^2)^		
	
	CG	50% HMEC	100% HMEC		T	L	Q
C4:0	4.53	4.30	4.33	0.21	0.24	0.21	0.63
C6:0	2.35	2.24	2.21	0.24	0.11	0.25	0.51
C8:0	1.26	1.25	1.19	0.08	0.24	0.65	0.32
C10:0	4.15^a^	3.68^b^	3.66^b^	0.15	0.01	0.72	0.41
C12:0	5.03^a^	4.65^b^	4.55^b^	0.16	0.03	0.63	0.55
C13:0	0.14	0.15	0.14	<0.01	0.09	0.78	0.34
C14:0	12.39^a^	11.20^b^	11.12^b^	0.15	0.04	0.31	0.61
C14:1	1.25	1.23	1.24	0.03	0.76	0.75	0.41
C15:0	1.25	1.31	1.26	0.03	0.18	0.87	0.48
C15:1	0.29	0.25	0.27	0.01	0.89	0.83	0.22
C16:0	26.89^a^	26.15^b^	26.21^b^	0.61	<0.01	0.64	0.24
C16:1	1.70	1.69	1.75	0.08	0.53	0.47	0.31
C17:0	0.91	0.84	0.86	0.79	0.18	0.86	0.55
C17:1	0.20	0.21	0.21	0.001	0.15	0.58	0.34
C18:0	7.65	7.61	7.59	0.28	0.44	0.47	0.37
trans-9C18:1	3.57	3.56	3.49	0.18	0.88	0.55	0.45
cis-9C18:1	19.56^b^	21.33^a^	21.59^a^	0.35	0.01	0.78	0.63
trans-6C18:2	0.36	0.33	0.34	0.02	0.30	0.86	0.41
cis-9cis-12C18:2	2.56	2.10	2.05	0.03	0.21	0.34	0.48
C18:3n-3	0.80	0.79	0.81	0.03	0.65	0.75	0.66
C20:0	0.67	0.61	0.65	0.05	0.62	0.68	0.34
SFA	65.22^a^	63.99^b^	63.77^b^	0.75	0.03	0.54	0.24
MUFA	26.57^b^	28.27^a^	28.55^a^	0.41	0.01	0.21	0.64
PUFA	3.72	3.22	3.20	0.11	0.85	0.64	0.38

HMEC, high moisture ear corn; FA, fatty acids; SEM, standard error of the mean; SFA, saturated fatty acids; MUFA, monounsaturated fatty acids; PUFA, polyunsaturated fatty acids.

1) CG, control group; 50% HMEC, replacing 50% steam-flaked corn with HMEC; 100% HMEC, replacing 100% steam-flaked corn with HMEC.

2) T, treatment; L, linear; Q, quadratic.

a,b Different lowercase superscripts within the same row indicate significant differences (p<0.05).

**Table 4:** Effect of HMEC on biochemical indices in primiparous dairy cows

Items	Treatment^1)^			SEM	p-value^2)^		
	
	CG	50% HMEC	100% HMEC		T	L	Q
ALT (U/L)	29.00	29.67	30.50	1.65	0.41	0.80	0.41
AST (U/L)	116.67	122.00	147.00	26.11	0.77	0.77	0.50
ALB (g/L)	37.72	37.42	37.89	0.45	0.54	0.81	0.35
GLU (mmol/L)	3.14	3.31	3.29	0.23	0.36	0.89	0.48
UREA (mmol/L)	6.42	5.80	5.88	0.43	0.32	0.83	0.33
CREA (μmol/L)	55.67	52.60	58.00	3.04	0.17	0.55	0.34
CHOL (mmol/L)	6.72	6.59	6.47	0.70	0.94	0.77	0.41
TG (mmol/L)	0.18	0.18	0.17	0.02	0.89	0.58	0.35
T3 (ng/mL)	2.96	2.84	2.62	0.16	0.14	0.88	0.44
T4 (ng/mL)	206.37	199.13	198.79	8.90	0.64	0.64	0.54

HMEC, high moisture ear corn; SEM, standard error of the mean; ALT, alanine aminotransferase; AST, aspartate aminotransferase; ALB, albumin; GLU, glucose; UREA, urea; CREA, creatinine; CHOL, cholesterol; TG, triglyceride.

1) CG, control group; 50% HMEC, replacing 50% steam-flaked corn with HMEC; 100% HMEC, replacing 100% steam-flaked corn with HMEC.

2) T, treatment; L, linear; Q, quadratic.

**Table 5:** Effect of HMEC on antioxidant status in serum of primiparous dairy cows

Items	Treatment^1)^			SEM	p-value^2)^		
	
	CG	50% HMEC	100% HMEC		T	L	Q
SOD (U/mL)	102.16^b^	104.68^ab^	110.13^a^	3.25	0.03	0.12	0.42
MDA (nmol/mL)	4.80^a^	4.52^ab^	4.08^b^	0.31	0.04	0.44	0.21
GSH-Px (U/mL)	950.50^b^	971.04^ab^	1,028.94^a^	24.45	0.03	0.21	0.39
T-AOC (U/mL)	9.33	9.25	9.99	0.38	0.24	0.84	0.64
CAT (U/mL)	7.23	7.37	7.87	0.33	0.17	0.64	0.51

HMEC, high moisture ear corn; SEM, standard error of the mean; SOD, superoxide dismutase; MDA, malondialdehyde; GSH-Px, glutathione peroxidase; T-AOC, total anti-oxidizing capability; CAT, catalase.

1) CG, control group; 50% HMEC, replacing 50% steam-flaked corn with HMEC; 100% HMEC, replacing 100% steam-flaked corn with HMEC.

2) T, treatment; L, linear; Q, quadratic.

a,b Different lowercase superscripts within the same row indicate significant differences (p<0.05).

**Table 6:** Effect of HMEC on immune responses in primiparous dairy cows

Items	Treatment^1)^			SEM	p-value^2)^		
	
	CG	50% HMEC	100% HMEC		T	L	Q
IgG (g/L)	10.50^b^	10.71^ab^	11.50^a^	0.46	0.04	0.35	0.44
IgA (g/L)	0.75	0.77	0.78	0.03	0.19	0.91	0.31
IgM (g/L)	2.66^b^	2.70^b^	2.94^a^	0.20	0.04	0.48	0.39
TNF-α (pg/mL)	45.52	43.31	44.30	18.91	0.99	0.51	0.41

HMEC, high moisture ear corn; SEM, standard error of the mean; IgG, immunoglobulin G; IgA, immunoglobulin A; IgM, immunoglobulin M; TNF-α, tumor necrosis factor-α.

1) CG, control group; 50% HMEC, replacing 50% steam-flaked corn with HMEC; 100% HMEC, replacing 100% steam-flaked corn with HMEC.

2) T, treatment; L, linear; Q, quadratic.

a,b Different lowercase superscripts within the same row indicate significant differences (p<0.05).
